# Microscale to Manufacturing Scale-up of Cell-Free Cytokine Production—A New Approach for Shortening Protein Production Development Timelines

**DOI:** 10.1002/bit.23103

**Published:** 2011-02-17

**Authors:** James F Zawada, Gang Yin, Alexander R Steiner, Junhao Yang, Alpana Naresh, Sushmita M Roy, Daniel S Gold, Henry G Heinsohn, Christopher J Murray

**Affiliations:** 1 Sutro Biopharma, Inc.310 Utah Ave. Suite 150, South San Francisco, California 94080; telephone: 650-888-6329; fax: 650-872-8924 e-mail: cmurray@sutrobio.com; 2 Rosebud Biotech3223 Santiago Street, San Francisco, California 94116

**Keywords:** cell-free protein synthesis, disulfide bond formation, transcription–translation

## Abstract

Engineering robust protein production and purification of correctly folded biotherapeutic proteins in cell-based systems is often challenging due to the requirements for maintaining complex cellular networks for cell viability and the need to develop associated downstream processes that reproducibly yield biopharmaceutical products with high product quality. Here, we present an alternative *Escherichia coli*-based open cell-free synthesis (OCFS) system that is optimized for predictable high-yield protein synthesis and folding at any scale with straightforward downstream purification processes. We describe how the linear scalability of OCFS allows rapid process optimization of parameters affecting extract activation, gene sequence optimization, and redox folding conditions for disulfide bond formation at microliter scales. Efficient and predictable high-level protein production can then be achieved using batch processes in standard bioreactors. We show how a fully bioactive protein produced by OCFS from optimized frozen extract can be purified directly using a streamlined purification process that yields a biologically active cytokine, human granulocyte-macrophage colony-stimulating factor, produced at titers of 700 mg/L in 10 h. These results represent a milestone for in vitro protein synthesis, with potential for the cGMP production of disulfide-bonded biotherapeutic proteins. Biotechnol. Bioeng. 2011; 108:1570–1578. © 2011 Wiley Periodicals, Inc.

## Introduction

Cell-free protein synthesis systems have distinct advantages over traditional in vivo methods for protein production (Endo and Sawasaki, 2006; Jermutus et al., 1998). The absence of a requirement to maintain cell viability allows optimization of the protein synthetic capacity of the cell-free extract to produce a single protein. The absence of a cell wall allows for addition of non-natural factors to the open system in order to manipulate transcription, translation, and folding to provide precise modulation of the protein expression process (Jewett and Swartz, 2004b). Cell-free systems derived from *Escherichia coli* have a long history as experimental tools for exploring the molecular biology of transcription and translation (Jewett et al., 2002; Noren et al., 1989; Spirin et al., 1988; Zubay, 1973) and have been utilized as a basis for developing in vitro protein production systems at small scales.

Cell-free protein production at the multigram and kilogram scale, an essential starting requirement for biotherapeutic production processes, has been hampered by the lack of scalable systems amenable to standard bioreactor configurations at large scale. Voloshin and Swartz (2008) reported production of 385 mg/L of soluble protein corresponding to a 3-disulfide bonded protein IGF-1 to 1-L scale. However, the fraction of correctly folded and functional protein was not reported, and different yields were reported using different reaction vessel designs. Consistent and robust performance at a wide range of scales is important for using a system as a general production technology and the time and cost of biologic drug discovery and production process development can be substantially reduced with systems that scale in a predictable and efficient manner from high-throughput screening to manufacturing.

The industrial scale application of cell-free protein synthesis for the production of recombinant proteins requires efficient and reproducible preparation of large volumes of active cell extracts (Liu et al., 2005), stable and cost-effective energy generating systems (Jewett and Swartz, 2004a), and the maintenance of stable pools of precursor molecules required for high-level protein synthesis. For example, the overall protein synthetic capacity of *E. coli* extracts has been increased by genetically engineering pathways responsible for recycling key amino acid precursor molecules, leading to the development of the *E. coli* strain KGK10 (Calhoun and Swartz, 2006; Knapp et al., 2007; Michel-Reydellet et al., 2004).

Here, we extend the *E. coli*-based system developed by Swartz and coworkers to show how scalable open cell-free synthesis (OCFS) can be optimized for high-level production of a multi-disulfide-bonded protein, granulocyte-macrophage colony-stimulating factor (rhGM-CSF), through detailed analysis and refinement of several process elements including: extract preparation and storage, optimization of synthetic gene sequences and redox parameters, process scale-up, and downstream product purification steps. We show that after full optimization, the OCFS system can produce active rhGM-CSF, a four-helix bundle human cytokine with two disulfide bonds required for functional activity. The system is linearly scalable and can achieve yields of rhGM-CSF of 700 mg/L in 10 h over ∼10^6^ range in volumes to 100 L. This linear scalability allows for rapid analysis of multiple parameters affecting transcription, protein synthesis, and folding in 24- or 96-well plates for high-throughput optimization of product and process parameters. Finally, the absence of a cell-lysis step after protein production, allows rapid and consistent protein purification optimization.

## Materials and Methods

### Cell Extract Prepared From Strain KGK10 at 200 L Scale

Cell extracts were prepared using a previously engineered K-12-derived *E. coli* strain KGK10 [A19 Δ*endA* Δ*tonA* Δ*speA* Δ*tnaA* met+ Δ*sdaA* Δ*sdaB* Δ*gshA* Δ*gor TrxB*_HAtag; (Knapp et al., 2007)]. KGK10 was cultured to mid-log phase [OD_595_∼45 OD, ∼140 g/L of cell wet weight (CWW)], using glucose and amino acid fed-batch fermentation at maximal growth rate of 0.7 h^−1^, essentially as described by (Zawada and Swartz, 2005), except that the glucose feed rate was increased manually to ensure excess glucose was present at the time of harvest. Fermentations were performed in a 200 L Sartorius D200 bioreactor equipped with pH and temperature control, four baffles, three six-bladed impellers, sampling tube, air sparger, pO_2_-probe, and exhaust gas analysis. Cells were pelleted by centrifugation at >14,000*g* for 45 min at 4°C two times using tubular bowl centrifuges (Model Z101; Carl Padberg, Schnell Zentrifugenbau GmbH, Lahr, Germany) or a disc-stack continuous centrifuge (Model CSC6 with hydrostop; GEA Westfalia GmbH, Oelde, Germany) with a maximum bowl speed of 12,000 rpm and a feed flow rate of 2.0–2.5 L/min, then resuspended and repelleted two times with S30 buffer and stored at −80°C.

The thawed cell-paste was suspended in 2 mL of S30 buffer per g CWW, cells were lysed in a homogenizer (EmulsiFlex-C55A; Avestin, Manheim, DE) at 20,000 psi and chilled to 0–4°C using a heat exchanger on the homogenizer outlet. Cell debris and insoluble components were removed by centrifugation at 12,000*g* for 45 min at 4°C two times. A typical 200 L fermentation yielded >1.1 L clarified extract/kg CWW with a total protein concentration of 20–25 g/L. For optimization of extract activity (“run-off reaction”), clarified extract samples (1 mL) were collected at various pre-incubation times at 25, 30, and 37°C, centrifuged at >15,000*g* to remove particulates, and then the supernatant was flash frozen in liquid nitrogen for subsequent analysis of protein synthesis activity, measured by rhGM-CSF production as described below. Once optimal pre-incubation conditions were identified, the extracts were activated at large scale and centrifuged at 14,000*g* for 35 min in tubular bowl centrifuges. The supernatant was frozen in liquid nitrogen and stored at −80°C in polyethylene bags or, for long-term stability studies, in Freeze-Pak Bio-container bags (60 mL; Charter Medical Ltd, Winston-Salem, NC), or at −40°C in cryostorage vials.

### Gene Expression Constructs

Expression of rhGM-CSF (GenBank Accession No. AAA52578.1) with an *N*-terminal methionine was under the control of the T7 promoter using added T7 RNA polymerase (RNAP) for transcription. Genes were designed using Biomax ProteoExpert (https://ssl.biomax.de/ProteoExpert/index.jsp) or DNA 2.0 GeneDesigner (https://www.dna20.com/genedesigner2/) algorithms (Welch et al. 2009) and were synthesized (DNA 2.0, Menlo Park, CA). Detailed methods for large-scale production of plasmid DNA and accessory proteins T7 RNAP and *E.coli* DsbC are described in Supplementary Methods.

### OCFS Protein Synthesis Reaction Conditions

Cell-free extracts were thawed to room temperature and incubated with 50 µM iodoacetamide for 30 min, as previously described (Knapp et al. 2007). OCFS reactions were run at 30°C for up to 10 h containing 30% (v/v) iodoacetamide-treated extract with 8 mM magnesium glutamate, 10 mM ammonium glutamate, 130 mM potassium glutamate, 35 mM sodium pyruvate, 1.2 mM AMP, 0.86 mM each of GMP, UMP, and CMP, 2 mM amino acids (1 mM for tyrosine), 4 mM sodium oxalate, 1 mM putrescine, 1.5 mM spermidine, 15 mM potassium phosphate, 100 nM T7 RNAP, 2–10 µg/mL plasmid DNA template, 1–10 µM *E. coli* DsbC. Reduced (GSH) and oxidized (GSSG) glutathione were added to a total concentration of ∼5 mM. The initial redox potential was calculated using the Nernst equation with *E*^0^ = −205 mV as the standard potential of the GSH/GSSG couple at 30°C and pH 7 (Wunderlich and Glockshuber, 1993).

Proteins produced at 250 µL scale or less were monitored by incorporation of l-[U-^14^C]-leucine (300 µCi/µmole; GE Life Sciences, Piscataway, NJ) by measuring the TCA-precipatible soluble and total protein in 24-well plates or Petri dishes without shaking (Voloshin and Swartz, 2005), or in 96-well plates at 30 µL scale (with shaking). Proteins were analyzed by reducing or non-reducing 12% SDS–PAGE gels with Sypro staining (Invitrogen, Carlsbad, CA) according to the manufacturer's recommendations and analyzed by autoradiography using a Storm 840 PhosphoImager.

At larger scales, concentrations of rhGM-CSF were determined using reversed phase HPLC (ZORBAX 300SB-C8 5 µm, 4.6 × 250 mm; Agilent, Santa Clara, CA) at 40°C with a flow rate of 1.5 mL/min in 100 mM triethylammonium acetate, pH 7.0 buffer. rhGM-CSF eluted 4 min into a 35–45.5%, acetonitrile gradient carried out over 7 min.

### OCFS Reaction Process Scale-up

OCFS reaction conditions were linearly scaled to 0.3 L in a 0.5 L stirred tank reactor (Biostat Q; B. Braun Biotech Inc., Bethlehem, PA), to the 4 L scale in a 10 L fermentor (ES10; B. Braun Biotech Inc.), and to the 100 L scale in a 200 L fermentor. Antifoam (1/12,000 v/v of 31R1 Pluronic® surfactant; BASF, Florham Park, NJ) was added, the pH maintained at 7.0 and dissolved oxygen concentration [O_2_] ∼20% by adjusting agitation speed and the [O_2_] in the sparge gas at 1 vvm and an initial ratio of 90% air to 10% O_2_. The 4 and 100 L scale reactions were pressurized at 5 psi to reduce foaming and 1 µg/mL ciprofloxacin was added to inhibit bacterial growth.

### Protein Purification

A portion of the rhGM-CSF was purified directly from the 100 L cell-free reaction as follows. Four liters of the 10 h cell-free reaction were diluted to 40 L with 10 mM sodium phosphate pH 6.5 and loaded onto 600 mL of DEAE Sepharose FastFlow resin (GE Life Sciences), washed with 10 L of 10 mM sodium phosphate pH 6.5, followed by gradient elution with 10 mM sodium phosphate, pH 6.5, 0.2 M NaCl over 30 column volumes. The DEAE purified product pool was concentrated 95-fold by tangential flow filtration with a 3 kD MW cutoff membrane (Pellicon 2 regenerated cellulose; Millipore, Billerica, MA). The concentrated product pool was then processed in multiple size exclusion chromatography runs using Sephacryl S-100 High-Resolution resin (GE Life Sciences) packed into two 1 L columns to give a combined column length of approximately 1 m. The overall recovery was 65%, and endotoxin and host cell protein levels were within specifications (Supplementary Methods). The activity of rhGM-CSF was assayed by the dose-dependent stimulation of the proliferation of human TF-1 cells as previously described (Goerke and Swartz, 2008).

## Results

### Cell Fermentation and Extract Preparation

The OCFS bioproduction process starts with high-density cell culture with fast growth rates in order to optimize the yield of ribosomes that is directly correlated with cell growth rate (Zawada and Swartz, 2006). We modified the short protocol of Liu et al. (2005) for extract production using fermentation under conditions of excess glucose at harvest, rather than glucose-limited fermentation. Several extract process improvements included, introduction of high-throughput centrifugation using a disc-stack centrifuge capable of processing hundreds of liters of cells, increased dilution of the cell suspension prior to homogenization to improve subsequent lysate clarification, as well as removal of the requirement for a dialysis step following the optimized pre-incubation protocol, as described below.

### Optimized Extract Activation and Storage Stability

An essential step in the preparation of the extract is the pre-incubation prior to the initiation of T7-based transcription and translation of protein product, sometimes referred to as a translation “run-off” procedure or pre-incubation step (Zubay, 1973). Although the exact nature of the biochemical steps associated with cell-extract pre-incubation are not known in detail, we chose to investigate the effect of time and temperature of the pre-incubation step on the synthesis activity of the clarified extract as measured by soluble rhGM-CSF production at small scale as described in Materials and Methods section.

**Figure 1:**
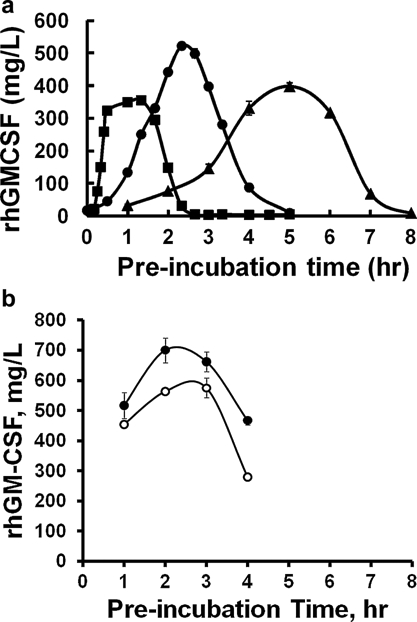
Protein synthesis activity is dependent on the time and temperature of extract pre-incubation. **a**: Aliquots of the cell-free extract were incubated at various times and temperatures (▪: 37°C, •: 30°C, and ▴: 25°C) to optimize the cell-free synthesis activity. **b**: Aliquots of the cell-free extract prepared using a tubular bowl centrifuge (•) or from cells harvested with large scale disc-stack continuous centrifugation, followed by lysate clarification in a tubular bowl centrifuge (○) were incubated at 30°C for various times. The concentration of rhGM-CSF produced after 5 h in a 60 µL scale reaction from these various pre-incubation conditions was determined by [^14^C]-leucine incorporation as described in the Materials and Methods section. CVs were ≤10% and the protein samples were ≥95% soluble for all time points.

Figure 1a shows that clarified extracts pre-incubated at 25, 30, or 37°C show differential amounts of rhGM-CSF are produced in the subsequent 5 h cell-free synthesis reaction. Optimal extract activation was achieved at 30°C using a 2.5-h pre-incubation period and this was confirmed with extract produced from cells harvested using large scale disc-stack centrifugation, as shown in Figure 1B. All subsequent reactions used extract pre-incubated under these activation conditions.

We also investigated the production capacity of activated extract after freezing and storage at −40°C and −80°C for periods up to 1 year (data not shown). Full rhGM-CSF production activity was retained, even after multiple freeze–thaw cycles, suggesting that active extract can be stored frozen for subsequent rapid protein production.

### Optimization of Gene Sequence Elements

**Figure 2:**
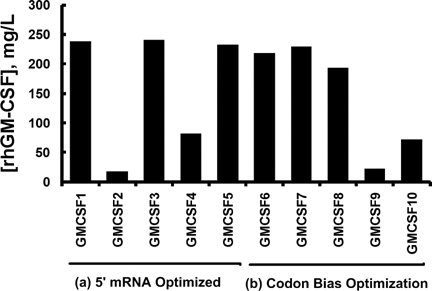
Yields of soluble rhGM-CSF for several gene sequences designed using (**a**) ProteoExpert 5′ mRNA optimization or (**b**) DNA 2.0 codon bias algorithms as described in the Materials and Methods section. The expression of soluble rhGM-CSF was measured by [^14^C]-leucine incorporation in 250 µL. CVs were ≤10%, for all constructs tested. The gene sequence of GM-CSF1 (Supplementary Methods) was used to optimize cell-free expression in scale-up experiments.

Synonymous gene mutations do not alter the encoded protein, but they can radically influence gene expression (Welch et al., 2009). To investigate how, we designed several gene constructs with synonymous codons designed to alter (a) the instability of the 5' mRNA sequence or (b) codon usage based on the algorithms described by Welch et al. (2009); see Supplementary Methods. As shown in Figure 2, synonymous codon variations lead to variable expression yields. We conclude that either gene sequence optimization method can give reasonable protein yields if multiple gene sequences are screened in the cell-free synthesis reaction. Higher throughput methods based on expression from linear DNA templates may allow a more rapid and extensive screening of this important optimization parameter (Son et al. 2006).

### Optimization of Disulfide Bond Formation

Conditions of the OCFS reaction can be directly manipulated to provide an optimized environment for both protein expression and folding. In contrast, the ability of *E. coli* to correctly fold recombinant proteins containing multiple disulfide bonds is limited, due the reducing environment of the cytosol and the limited capacity for co-translational folding (Baneyx and Mujacic, 2004; Endo and Sawasaki, 2006; Jermutus et al., 1998; Kawasaki et al., 2003; Kolb et al., 2000; Maier et al., 2005; Yang et al., 2004).

**Figure 3:**
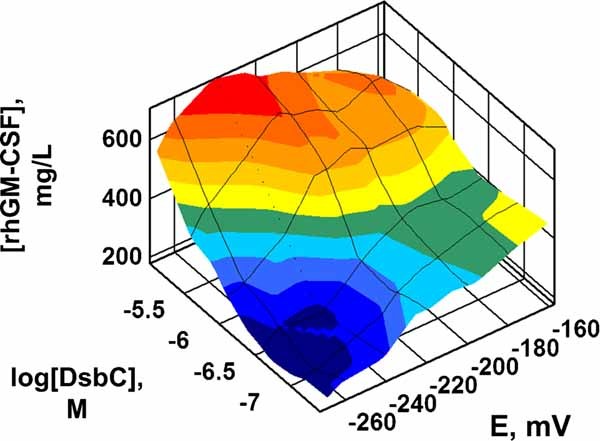
Combinatorial optimization of protein folding in the OCFS system. Response surface of redox potential and DsbC concentration on soluble expression of rhGM-CSF. The expression of soluble rhGM-CSF was measured by [^14^C]-leucine incorporation in a 96-well plate for IAM-treated extract as a function of the initial redox potential of added [GSSG]_tot_ = 5 mM and DsbC concentration. For reference, the redox potential of the *E. coli* cytosol is −270 mV.

Figure 3 shows that the optimal conditions for production of soluble, folded rhGM-CSF are controlled both by the redox potential and the concentration of added *E. coli* disulfide isomerase, DsbC, that catalyzes disulfide bond rearrangement (Yang et al., 2004). These experiments were carried out in 96-well microtiter scale as measured by the amount of soluble rhGM-CSF produced. Under optimized redox conditions the ratio of soluble:total GM-CSF was ≥95%, consistent with correctly folded disulfide bonds, subsequently confirmed by MS analysis of rhGM-CSF produced under these optimized conditions.

Optimization of protein synthesis yield required us to balance efficient translation, which requires reducing conditions (Traut and Haeeni, 1967; Yang et al., 2004), with protein folding of correct disulfide bonds that requires a more oxidizing environment. DsbC must be maintained in a reduced state to be able to catalyze the rearrangement of non-native disulfide bonds but has a non-specific folding chaperone activity as well. The high DsbC concentrations required for efficient soluble protein yield suggests that the folding chaperone activity of DsbC dominates, although the drop-off in soluble yield under more oxidizing conditions is consistent with a requirement for reduced DsbC for efficient disulfide isomerase activity (Ryabova et al., 1997).

### OCFS is Linearly Scalable from Microscale to Large Scale Reaction

**Figure 4:**
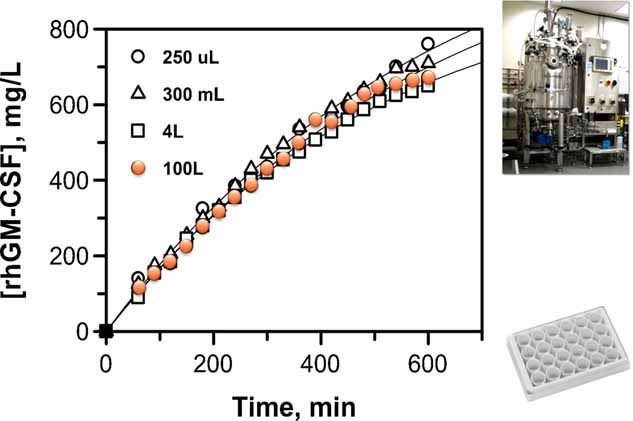
The rate of soluble rhGM-CSF production is independent of scale from plates, to stirred tank reactors, to large-scale bioreactors. The initial slopes corresponds to a translation rate of ∼1 peptide bond per second per ribosome. The concentrations of rhGM-CSF were determined by [^14^C]-leucine incorporation at 250 µL scale, and by RP HPLC analysis at larger scales, as described in the Materials and Methods section. CVs were ≤10% and the proteins were ≥95% soluble for all time points. Lines are shown for visual purposes only.

We used the optimization parameters described above to define process parameters for scale-up, without changes in volumetric performance of the system at all scales. Figure 4 shows that the OCFS process exhibits linear scalability over a ∼10^6^ change in reaction volume for rhGM-CSF, with yields of 700 mg/L after 10 h. This high yield of fully soluble protein makes the requirement for refolding of rhGM-CSF produced in normal bacterial fermentation processes (Berges et al., 1996; Libby et al., 1987) unnecessary and simplifies subsequent downstream processing (vida infra).

### OCFS is Efficient

The overall efficiency of the OCFS system compares favorably with corresponding efficiencies for protein translation in vivo (Table I). Elongation rates of ∼1 amino acid (aa) per second per ribosome are about 20-fold higher than comparable eukaryotic cell-free systems (Endo and Sawasaki, 2006; Kawasaki et al., 2003), but smaller than the rate of 10–20 aa s^−1^ in vivo for rapidly growing *E coli*. Similarly, initiation rates in vitro are smaller than estimated rates in vivo. In vivo expression of heterologous proteins must compete with the protein synthesis required for cell growth and viability, so that the effective heterologous protein synthesis rate in vivo may be lower. The slower translation rate may also aid in efficient co-translational folding of rhGM-CSF in the presence of the directly added DsbC chaperone.

**Table I:** Ribosomal translation in vivo and in vitro^a^

	Translation efficiency			
			
Translation system	Initiation rate^b^ (s^−1^)	Elongation rate (AA s^−1^ ribosome^−1^)	Productive synthesis time (h)	References
*E. coli* cell	0.5	10–20^c^	5–24	Gromadski and Rodnina (2004); Kierzek et al. (2001); Parker (1989)
OCFS system	0.0015^d^	∼1^e^	∼10	This work
Cytomim system	0.017	∼1	∼4	Underwood et al. (2005); Voloshin and Swartz (2008)
PURE system		2	1	Shimizu et al. (2001)
Wheat germ extract (WGE)		0.05^f^	∼2	Kawasaki et al. (2003)

a At 37°C, unless otherwise indicated.

b Initiation rate is the rate of ribosome clearance, as described by Kierzek et al. (2001).

c Translation rate estimated based on in vivo data for *E. coli* in minimal or rich medium or estimated from single-turnover experiments.

d Determined from the kinetics of GFP production using the model of Kierzek et al., unpublished results.

e Translation rate at 30°C derived from initial translation rate: 150 mg/L/h (Fig. 4), rhGM-CSF MW = 15 kD, and an active ribosome concentration = 300 nM (Supplementary Fig. S5).

f Batch mode scFv synthesis using eukaryotic wheat germ extract (WGE) translation. The elongation rate for a 26 kD scFv is estimated from the observed maximal synthesis rate of 18 µg/mL/h total protein, 85% soluble protein, a WGE *A*_260_ = 42; where 1 *A*_260_ = 20 nM eukaryotic ribosomes. The translation elongation rate is 10 times slower in eukaryotes.

The yields achieved here were obtained using standard stirred tank bioreactors in batch mode without feeding supplemental reagents over the 10 h production process. OCFS is able to produce protein for longer times compared to other in vitro protein synthesis systems in a comparable batch mode (Table I), due to the ability to maintain steady state levels of NTPs generated by active oxidative phosphorylation pathways. As alternatives, continuous or semi-continuous formats of cell-free protein synthesis that require a supply of high-energy NTP substrates and a reservoir to remove inhibitory byproducts have been developed (Endo and Sawasaki, 2006; Jewett and Swartz, 2004b; Spirin et al., 1988). Although continuous-feed approaches increase the duration of protein synthesis, they are not scalable, are expensive, and are unwieldy due to membrane fouling for both high-throughput screening and large-scale production.

### OCFS Allows Efficient Downstream Protein Purification

**Figure 5:**
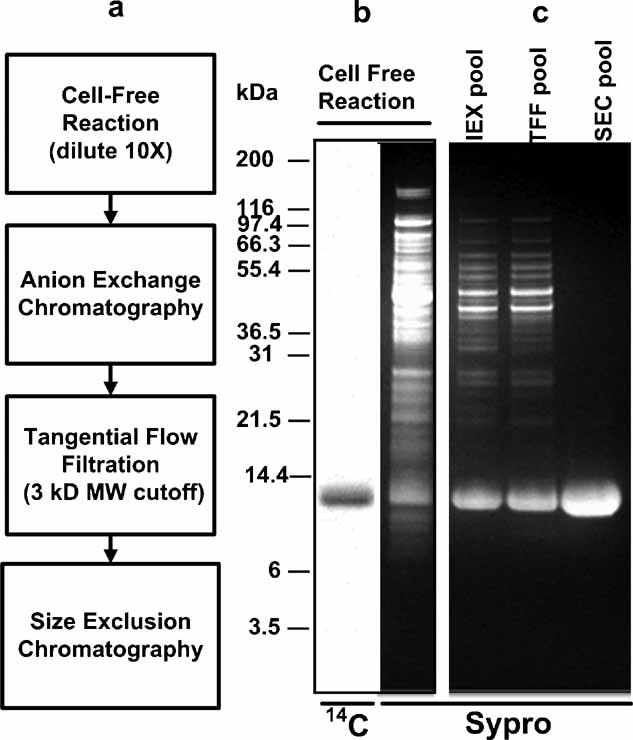
Analysis of process purification steps used in the production of pharmaceutical-grade rhGM-CSF. **a**: Process flow diagram for protein purification (**b**) Cell-free reaction product pool was visualized, using non-reducing SDS–PAGE, by ^14^C autoradiography of leucine incorporation at 5 h that measures only protein produced, or by Sypro staining of all proteins in the extract at 10 h. Autoradiography indicates incorporation of ^14^C-leucine corresponding to the molecular weight of rhGM-CSF and confirms that only rhGM-CSF was produced because the T7 RNAP limits transcription to the T7 promoter on the plasmid added to the reaction, with no evidence for aberrant protein products produced from native RNAP transcription. **c**: Non-reducing SDS–PAGE of the process purification pools. **Lane 1**: Anion exchange pool. **Lane 2**: Tangential flow filtration pool. **Lane 3**: SEC final product pool.

In engineering an effective large-scale production method for biologic products it is important to design a system that uses standard downstream process equipment and reduces the requirement for multiple manipulations of the product during the purification process. We therefore developed a streamlined two-column process for purification of rhGM-CSF directly from the cell-free production extract, as outlined in Figure 5.

Because the cell-free reaction product pool contains soluble, correctly folded protein product and is substantially reduced in endotoxin levels and other cellular debris, it could be loaded directly onto an anion exchange resin, without significant degradation in column performance during multiple purification runs. Subsequent concentration by tangential flow filtration and removal of host cell proteins by size exclusion chromatograpy (SEC)-yielded fully bioactive product with 65% overall recovery of high purity rhGM-CSF.

**Figure 6:**
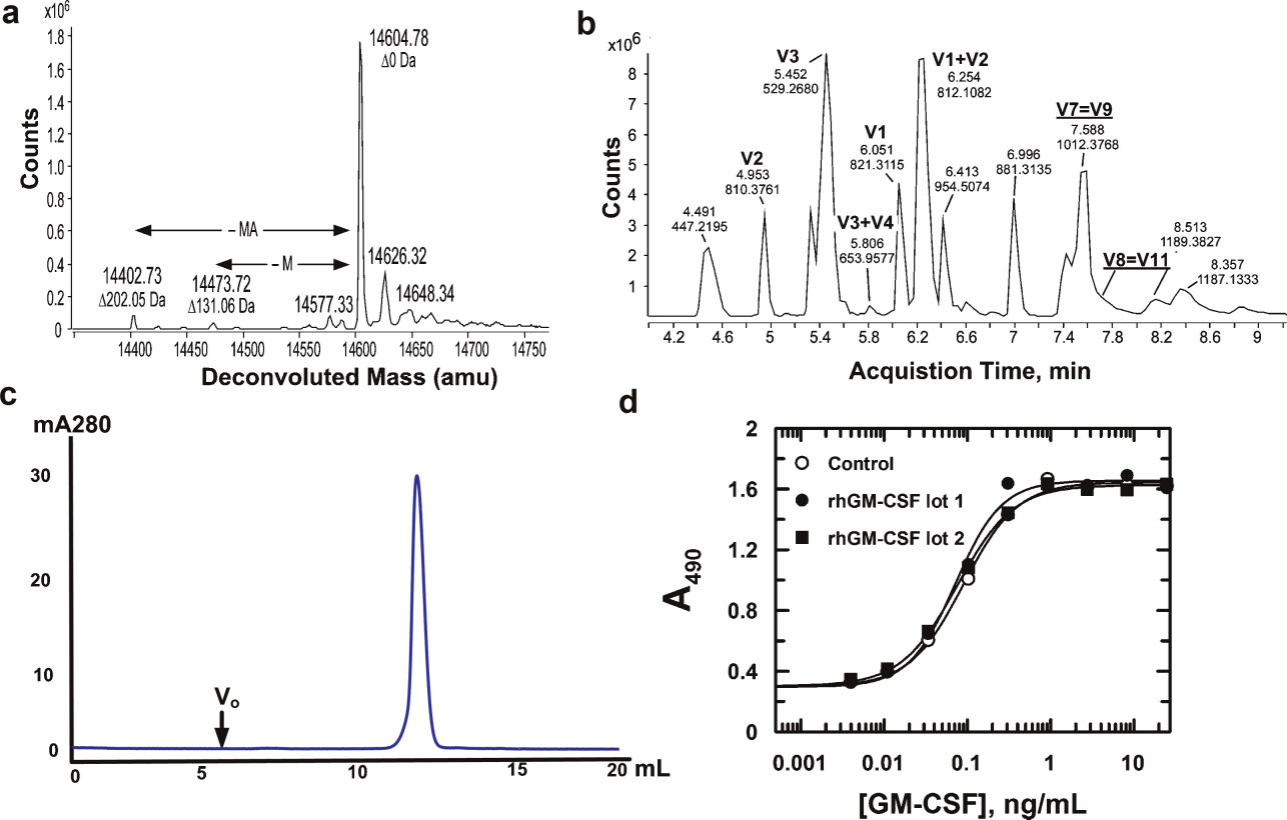
rhGM-CSF is correctly folded and functional. **a**: Liquid chromatography–Mass spectrometry analysis of intact mass of 14604.78 Da corresponds to the expected average neutral mass of 14604.75 Da. Two minor product related impurities were observed corresponding to removal of the first two amino acids (-M and -MA). These peak intensities do not correspond accurately to the relative amount of product related impurities. **b**: Base peak chromatogram of a V8 protease digestion of rhGM-CSF. The two peptides with disulfide bridges labeled V7 = 9 and V8 = 11 (underlined) coincide with the theoretical masses with correct disulfide bridge formation. The V7 = 9 linkage was confirmed by tandem MS and the V8 = 11 by accurate mass. Other observed V8 digest peptides are marked accordingly. Digest peptides denoted with a plus sign indicate a missed cleavage. **c**: Analytical size exclusion chromatogram of purified rhGM-CSF on a Tosoh TSKgelG3000SWXL column, flow rate 0.5 mL/min. **d**: The effective dose 50, ED_50_ <0.1 ng/ml of rhGM-CSF induced proliferation of human TF-1 cells, corresponds to a specific activity of 14 × 10^6^ IU/mg, comparable to commercially available *E. coli*-derived rhGM-CSF protein as a positive control.

To assess protein quality produced by the OCFS system, we used a series of common comparability protocols in biopharmaceutical manufacturing (Chirino and Mire-Sluis, 2004), as summarized in Figure 6 and Supplementary Methods. We confirmed the absolute mass and correct disulfide bonding patterns by LC-MS, and LC-MS/MS sequencing, SEC was used to determine purity, and the biological activity of rhGM-CSF was determined in a cell-based proliferation assay. The purity achieved could be considered suitable for therapeutic use but could also be improved further using three-step purification. We conclude that OCFS can readily produce proteins of pharmaceutical grade quality at commercially relevant yields and scales.

## Discussion

Several cell-based secretion systems produce biologically active GM-CSF in *E. coli* (Berges et al., 1996; Libby et al., 1987), yeast [50–60 mg/L; (Price et al., 1987)], *Aspergillus niger* [0.64 µg/L;(Kim et al. 2000)], *Pichia pastoris* (200–250 mg/L; (Sainathan et al., 2005) and 130 mg/L; [Bhatacharya et al., 2007)], and insect cells (11–45 mg/L;[Chiou and Wu, 1990)]. In general, substantial losses of product due to refolding, filtration, and extensive column chromatography steps are required to isolate homogenously pure product in these systems. Cell-free protein synthesis of GM-CSF as well as other small, disulfide-bonded proteins has been described previously at small scale (Goerke and Swartz, 2008; Kawasaki et al., 2003; Ryabova et al., 1997; Son et al., 2006; Yang et al., 2004).

Our approach represents an advance towards a robust (>95% correctly folded multi-disulfide-bonded protein), high yield (700 mg/L protein over 10 hr), linearly scalable (∼10^6^ fold), and high-recovery (>65%) system for industrial production of disulfide-bonded proteins such as GM-CSF. Using the open nature of cell-free synthesis we optimized several factors affecting transcription, translation, and folding to predictably identify conditions for scalable production of a disulfide-bonded protein to the 100 L scale. In this in vitro synthetic biology-based system, experimental data built from an understanding of the energy source as well as transcription, translational, and protein folding components are sufficient to guide design and testing of protein expression conditions in a microtiter plate format with predictable properties at any scale. More complex secondary effects associated with in vivo gene expression and regulatory networks are avoided. For example, in T7-based systems in vivo, transcription, translation, and folding are uncoupled. The uncoupling mediated by T7 RNAP may in turn mediate protein aggregation. However, after full optimization of plasmid, T7 RNAP, and redox conditions, >95% of the protein produced is soluble, there is no evidence of significant misfolded aggregates formed as measured by non-reducing SDS–PAGE autoradiography (Fig. 5b), or detected in the final product (Fig. 6c).

Previous optimization strategies for cell-free protein expression have sampled parameters sparsely and thus cannot be generalized to the more complex, finely balanced networks of protein synthesis and folding described here. Instead, we found that one must first assemble and experimentally characterize interacting networks of myriad system components at small scale to create generalizable response surfaces such as those in Figures 1 and 3 with quantitative predictive capabilities for process scale-up.

The OCFS system uses a K-12-derived production strain as the basis for a cell-line banked under cGMP conditions. Multiple protein products can now be produced on an as needed basis from the same stored frozen extract produced from this strain. For example, similar scalable results to 4 L have been obtained for a single-chain antibody fragment (scFv), anti-IL-23 scFv (1 g/L in 10 h) and an anti-IL13Rα1 Fab antibody with five disulfide bonds (300 mg/L in 9 h; manuscript in preparation). Use of a single cell-line coupled with the speed of process optimization inherent in the linearly scalable OCFS protein production system should allow for decreases in the timelines for biopharmaceutical protein production and process development.

We have developed the OCFS production process using standard microbial fermentation and process equipment known to scale to thousands of liters under cGMP standards. Additional efforts to improve this production process toward cGMP compliance include (a) developing methods for sterile filtration of the extract, (b) establishing extract process production reproducibility, (c) establishing the translational fidelity of OCFS produced proteins (Heibeck et al., manuscript in preparation), and (d) accurate cost-modeling of the reagents and other process variables. Finally, we aim to develop a molecular understanding of the limiting factors accounting for the time and temperature dependence of extract activation shown in Figure 1, by quantitative LC-MS-based profiling of proteins and metabolites (Roy and Becker, 2007), in order to improve protein production yields further.

The OCFS system is limited to producing proteins with only a few post-translational modifications such as disulfide bonds and proteolytic processing (Son et al., 2006); other therapeutic protein classes such as glycosylated proteins are currently outside the scope of OCFS at large scale. However, OCFS has the potential for synthesis of proteins containing site-specifically incorporated non-natural amino acids (Noren et al., 1989) without the requirement for orthogonal tRNA synthetase-tRNA pairs or limitations to cellular uptake of non-natural amino acids (Antonczak et al., 2009). This will allow the engineering of novel post-translationally modified proteins using the scalable OCFS system.
