# Supplementary material for: Microscale to Manufacturing Scale-up of Cell-Free Cytokine Production—A New Approach for Shortening Protein Production Development Timelines
Source: Biotechnol Bioeng. 2011 Feb 17;108(7):1570–8. doi: 10.1002/bit.23103 (PMC3128707; doi:10.1002/bit.23103)
Supplement: Supplementary file 1 [file bit0108-1570-SD1.doc]

# Supplementary Methods

for

Microscale to Manufacturing Scale-up of Cell-Free Cytokine Production – a New approach for shortening Protein Production Development Timelines

James F. Zawada^1^, Gang Yin^1^, Alexander R. Steiner^1^, Junhao Yang^1^, Alpana Naresh^1^, Sushmita M. Roy^1^, Henry G. Daniel S. Gold, Henry H. Heinsohn^1^ & Christopher J. Murray^1^

T7 Plasmid Design.

The engineered high copy T7-based plasmid pYD317-GM1 (**Figure S1**) contains kanamycin resistance gene, an engineered high-copy ColE1 origin of replication, the T7 gene 10 translational enhancer (Olins et al. 1988), the Shine-Dalgarno (SD) sequence as a binding site for prokaryotic ribosomes (RBS) with an optimum distance to the start AUG codon (Chen et al., 1994), and a strong T7 promoter sequence driving transcription. The full sequence from the T7 promoter to the ATG start codon is 5’TAATACGACTCACTATAGGGAGACCACAACGGTTTCCCTCTAGAAATAATTTTGTTTAACTTTAAGAAGGAGATATACAT**ATG**-3’. Genes coding for GenBank Accession No. AAA52578.1 were cloned in frame with the ATG start codon using *Nde1* and *Sal1* cloning sites. A strong T7 transcription terminator hairpin prevents elongated transcripts and 3’-terminal exonucleolytic degradation of the mRNA (Ahn et al. 2008). DH5α transformed E. coli containing pYD317-GM1 was purified from 1 kg of cell-paste using a modification of the method of Zhang et al (Zhang et al. 2003) using 500 g of Fractogel DEAE resin (Merck KgaA) to yield 2.6 g of plasmid. pYD317-based plasmids typically run as three bands on DNA gels (**Figure S1**).

Gene Sequence Optimization

Genes were designed using Biomax ProteoExpert (<https://ssl.biomax.de/ProteoExpert/index.jsp>) or DNA 2.0 GeneDesigner (<https://www.dna20.com/genedesigner2/>; (Welch et al. 2009)).They use different approaches to optimize genes. The gene candidates were synthesized, cloned into pYD317 vector using *Nde1/Sal1*, transformed into DH5α, and plasmids were purified, and tested with the cell-free system at small-scale. It should be noted that Proteoexpert changes the first six codons only, while DNA2.0 optimizes the whole gene. **Figure S2** summarizes the aligned rhGM-CSF sequences (note the absence of the ATG start codon). All scale-up work was done with pYD317-GM1 as described in the text.

**Figure S1. (a)** Plasmid Map of pYD317-GMCSF5. **(b)** DNA gel electrophoresis of pYD317-GMCSF5 plasmid. Lane 1: Marker (bp ladder), Lane 2: 1 μg plasmid.

**a**

**b**

**
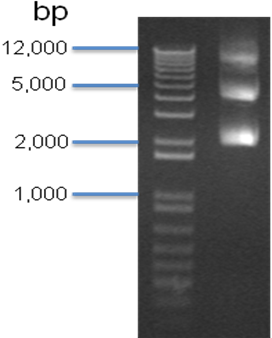
**

**Fig S2. Multiple sequence alignment of gene optimized rhGM-CSF mutants.**

1 100

GMCSF1 (1) GCACCAGCACGTAGTCCAAGCCCCAGCACGCAGCCCTGGGAGCATGTGAATGCCATCCAGGAGGCCCGGCGTCTCCTGAACCTGAGTCGTGACACTGCTG

GMCSF2 (1) GCACCAGCACGTTCACCAAGCCCCAGCACGCAGCCCTGGGAGCATGTGAATGCCATCCAGGAGGCCCGGCGTCTCCTGAACCTGAGTCGTGACACTGCTG

GMCSF3 (1) GCACCAGCACGTAGTCCTAGCCCCAGCACGCAGCCCTGGGAGCATGTGAATGCCATCCAGGAGGCCCGGCGTCTCCTGAACCTGAGTCGTGACACTGCTG

GMCSF4 (1) GCACCAGCACGCAGTCCAAGCCCCAGCACGCAGCCCTGGGAGCATGTGAATGCCATCCAGGAGGCCCGGCGTCTCCTGAACCTGAGTCGTGACACTGCTG

GMCSF5 (1) GCACCAGCTCGTTCACCTAGCCCCAGCACGCAGCCCTGGGAGCATGTGAATGCCATCCAGGAGGCCCGGCGTCTCCTGAACCTGAGTCGTGACACTGCTG

GMCSF6 (1) GCACCAGCACGCAGCCCAAGCCCAAGCACGCAACCATGGGAGCACGTCAATGCAATTCAAGAGGCACGCCGCCTGCTGAATCTGAGCCGCGACACGGCAG

GMCSF7 (1) GCCCCGGCACGTAGCCCAAGCCCATCCACCCAACCATGGGAGCACGTAAATGCAATTCAAGAGGCCCGTCGATTGCTGAATTTGAGCCGAGACACGGCGG

GMCSF8 (1) GCACCGGCCCGTAGCCCTAGCCCAAGCACCCAACCGTGGGAGCATGTTAACGCGATCCAGGAAGCCCGTAGATTGCTGAACCTGAGCCGTGACACCGCAG

GMCSF9 (1) GCACCGGCTCGTAGCCCAAGCCCATCCACGCAACCTTGGGAGCATGTTAACGCGATCCAGGAAGCCCGTAGATTGCTGAACCTGAGCCGTGACACCGCAG

GMCSF10 (1) GCACCGGCTCGCAGCCCGTCCCCAAGCACCCAACCATGGGAACATGTCAATGCCATCCAAGAGGCACGCCGTCTGCTGAACCTGTCTCGCGATACTGCGG

101 200

GMCSF1 (101) CTGAGATGAATGAAACAGTAGAAGTCATCTCAGAAATGTTTGACCTCCAGGAGCCGACCTGCTTACAGACCCGCCTGGAGCTGTACAAGCAGGGCCTGCG

GMCSF2 (101) CTGAGATGAATGAAACAGTAGAAGTCATCTCAGAAATGTTTGACCTCCAGGAGCCGACCTGCTTACAGACCCGCCTGGAGCTGTACAAGCAGGGCCTGCG

GMCSF3 (101) CTGAGATGAATGAAACAGTAGAAGTCATCTCAGAAATGTTTGACCTCCAGGAGCCGACCTGCTTACAGACCCGCCTGGAGCTGTACAAGCAGGGCCTGCG

GMCSF4 (101) CTGAGATGAATGAAACAGTAGAAGTCATCTCAGAAATGTTTGACCTCCAGGAGCCGACCTGCTTACAGACCCGCCTGGAGCTGTACAAGCAGGGCCTGCG

GMCSF5 (101) CTGAGATGAATGAAACAGTAGAAGTCATCTCAGAAATGTTTGACCTCCAGGAGCCGACCTGCTTACAGACCCGCCTGGAGCTGTACAAGCAGGGCCTGCG

GMCSF6 (101) CAGAGATGAATGAGACGGTCGAGGTCATTAGCGAGATGTTCGACCTGCAAGAGCCAACGTGCCTGCAAACGCGCCTGGAGCTGTACAAGCAAGGCCTGCG

GMCSF7 (101) CAGAGATGAACGAGACGGTAGAGGTGATTAGCGAGATGTTCGATCTGCAAGAGCCGACCTGCTTGCAAACCCGTCTTGAGCTTTACAAGCAGGGTCTGCG

GMCSF8 (101) CAGAAATGAACGAGACGGTGGAAGTTATTAGCGAAATGTTCGACCTGCAGGAGCCGACGTGTCTGCAAACCCGTCTGGAGCTGTACAAACAGGGTCTGCG

GMCSF9 (101) CAGAAATGAACGAGACGGTGGAAGTTATTAGCGAAATGTTCGACCTGCAGGAGCCTACGTGTCTGCAAACCCGTCTGGAGCTGTACAAACAGGGTCTGCG

GMCSF10 (101) CGGAAATGAACGAGACTGTCGAAGTGATTAGCGAAATGTTTGATCTGCAGGAGCCTACGTGCCTGCAGACCCGTCTGGAACTGTATAAACAAGGTCTGCG

201 300

GMCSF1 (201) GGGCAGCCTCACCAAGCTCAAGGGCCCCTTGACCATGATGGCCAGCCACTACAAGCAGCACTGCCCTCCAACCCCGGAAACTTCCTGTGCAACCCAGATT

GMCSF2 (201) GGGCAGCCTCACCAAGCTCAAGGGCCCCTTGACCATGATGGCCAGCCACTACAAGCAGCACTGCCCTCCAACCCCGGAAACTTCCTGTGCAACCCAGATT

GMCSF3 (201) GGGCAGCCTCACCAAGCTCAAGGGCCCCTTGACCATGATGGCCAGCCACTACAAGCAGCACTGCCCTCCAACCCCGGAAACTTCCTGTGCAACCCAGATT

GMCSF4 (201) GGGCAGCCTCACCAAGCTCAAGGGCCCCTTGACCATGATGGCCAGCCACTACAAGCAGCACTGCCCTCCAACCCCGGAAACTTCCTGTGCAACCCAGATT

GMCSF5 (201) GGGCAGCCTCACCAAGCTCAAGGGCCCCTTGACCATGATGGCCAGCCACTACAAGCAGCACTGCCCTCCAACCCCGGAAACTTCCTGTGCAACCCAGATT

GMCSF6 (201) CGGCAGCCTGACGAAGCTGAAGGGCCCACTGACGATGATGGCAAGCCACTACAAGCAACACTGCCCACCAACGCCAGAGACGAGCTGCGCAACGCAAATT

GMCSF7 (201) AGGCTCATTGACGAAGTTGAAAGGTCCGCTGACGATGATGGCCAGCCATTACAAACAACACTGTCCGCCAACCCCGGAGACGAGCTGCGCAACCCAAATT

GMCSF8 (201) TGGTAGCCTGACCAAACTGAAGGGCCCGCTGACCATGATGGCGAGCCACTATAAACAACATTGCCCGCCGACCCCGGAGACGTCCTGCGCGACCCAAATC

GMCSF9 (201) TGGTAGCCTGACCAAACTGAAGGGCCCACTGACCATGATGGCGAGCCACTATAAACAACATTGCCCGCCCACCCCTGAGACGTCCTGCGCGACCCAAATC

GMCSF10 (201) TGGTTCTCTGACCAAGCTGAAAGGTCCGCTGACCATGATGGCAAGCCACTATAAACAGCACTGCCCACCGACTCCGGAGACTTCTTGTGCCACTCAAATC

301 381

GMCSF1 (301) ATCACCTTTGAAAGTTTCAAAGAGAACCTGAAGGACTTTCTGCTTGTCATCCCCTTTGACTGCTGGGAGCCAGTCCAGGAG

GMCSF2 (301) ATCACCTTTGAAAGTTTCAAAGAGAACCTGAAGGACTTTCTGCTTGTCATCCCCTTTGACTGCTGGGAGCCAGTCCAGGAG

GMCSF3 (301) ATCACCTTTGAAAGTTTCAAAGAGAACCTGAAGGACTTTCTGCTTGTCATCCCCTTTGACTGCTGGGAGCCAGTCCAGGAG

GMCSF4 (301) ATCACCTTTGAAAGTTTCAAAGAGAACCTGAAGGACTTTCTGCTTGTCATCCCCTTTGACTGCTGGGAGCCAGTCCAGGAG

GMCSF5 (301) ATCACCTTTGAAAGTTTCAAAGAGAACCTGAAGGACTTTCTGCTTGTCATCCCCTTTGACTGCTGGGAGCCAGTCCAGGAG

GMCSF6 (301) ATTACGTTCGAGAGCTTCAAGGAGAATCTGAAGGACTTCCTGCTGGTCATTCCATTCGACTGCTGGGAGCCAGTCCAAGAG

GMCSF7 (301) ATTACCTTCGAGTCATTCAAGGAGAATCTTAAAGACTTTCTGCTGGTCATTCCGTTCGACTGCTGGGAGCCAGTGCAAGAG

GMCSF8 (301) ATTACCTTCGAGTCGTTTAAGGAAAATCTGAAAGACTTTCTGCTGGTGATCCCGTTCGACTGTTGGGAGCCGGTCCAGGAA

GMCSF9 (301) ATTACCTTCGAGTCGTTTAAGGAAAATCTGAAAGACTTTCTGCTGGTGATCCCGTTCGACTGTTGGGAGCCGGTCCAGGAA

GMCSF10 (301) ATCACCTTTGAATCCTTTAAGGAAAATCTGAAAGACTTTCTGCTGGTGATCCCATTCGATTGTTGGGAACCTGTACAAGAG

Anion Exchange-HPLC

Column: Dionex WAX-10 (4.6 mm x 250 mm)

Buffer A: 20 mM bis-Tris, pH 5.8

Buffer B: 20mM bis-Tris, 500 mM NaCl, pH 5.8

Flow rate: 1.5mL/min


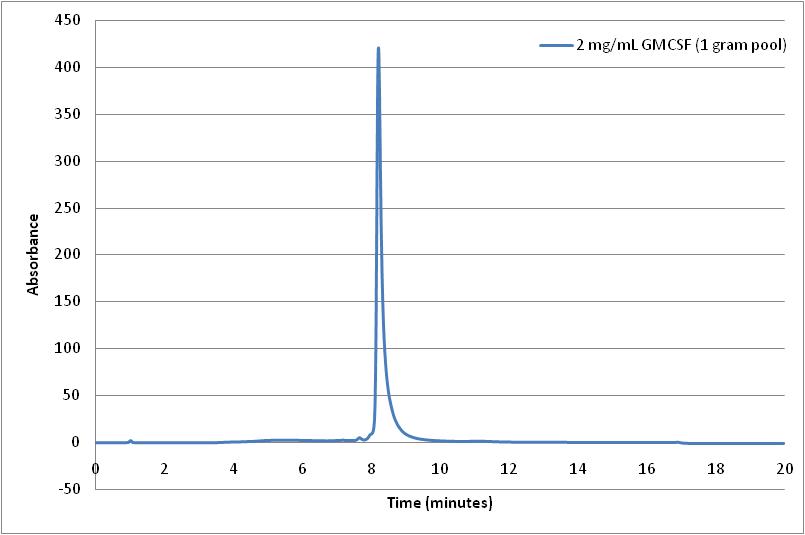
Elution: 0-2min: 0% B; 2-15min gradient: 0% B to 100%; 15-15.5 min: 100%B; 15.5-20 min: 0% B

**Figure S3** Anion Exchange HPLC analysis of purified rhGM-CSF**.**

Endotoxin Concentration Analysis.

Endotoxin units (EU) were measured using the Limulus Amebocyte Lysate Endosafe KTA kit (Charles River Laboratories) including: Limulus Amebocyte Lysate (LAL; Product #R19000), *E.coli* Control Standard Endotoxin (CSE; Product #E120), LAL Reagent Water (Product #W120), Endosafe Microtiter Plate (Product #M9005) following procedures specified by the manufacture. The concentration of product was determined independently of this assay by RP-HPLC and calculation of endotoxin was based on the standard curve, corrected for dilution, and yielded endotoxin levels of < 2.2 EU/mg rhGM-CSF.

*E Coli* Host Cell Protein Assay*.*

The measurement of *E.coli* Host Cell Protein (HCP) was accomplished using the Enzymatic Assay for the Measurement of E.coli Host Cell Proteins Kit (Cygnus Technologies Product #F410) with Sample Diluent Buffer (Cygnus Technologies Product #I028). The procedure used was based on the kit manual, with minor modifications. HCP concentrations were calculated based on the standard curve, corrected for dilution, and yielded <18 ng HCP/mg rhGM-CSF

DNA/RNA Quantification Assay*.*

The concentration of contaminating DNA/RNA was determined using a Picogreen Assay, yielding < 0.4 ng DNA/RNA per mg rhGM-CSF.

Liquid chromatography-Mass spectrometry

Liquid chromatography-Mass spectrometry were run with a Agilent 1200 Series Binary Pump coupled to an Agilent 6520 Accurate Mass Q-TOF LC-MS and analyzed using MassHunter software. The intact mass spectrum was measured via direct injection in 0.1% formic acid with no separator column at 0.4 mL/min.

Peptide mapping of rhGM-CSF was performed by digestion with V8 protease at 1:20, enzyme: substrate, added at t = 0 h and 15 h for a total of 22 h at 37 °C. After digestion samples were acidified to 0.5% formic acid and snap frozen. The digest solution was separated over an Agilent Zorbax SB-Aq column with an acetonitrile gradient from 0-65% over 10 min. Tandem mass spectra were acquired automatically choosing the two 2+ ions for each parent ion scan. Collision energy for tandem MS was calculated with the formula (3.7 x m/z)/100 + 2.5.

Upon reduction of the purified intact product with DTT, the mass spectrum shows an increase in protonation sites, suggesting opening up of the folded structure (**Figure S4**). The increase in protonation sites is demonstrated by the shifting of the mass-to-charge ratios of observed protein molecules, upon reduction, to the left of the mass spectrum due to an increase in charge. These data show that the purified rhGM-CSF is well-folded and in its native state.

Figure S4 Intact mass of rhGM-CSF analyzed by electrospray ionization mass spectrometry before and after DTT reduction.

Field Flow Fractionation (FFF) measurement of ribosome concentration

Asymmetrical Field flow fractionation of ribosome concentrations in the cell-free extract was performed with a AF2000 MT FFF device and an on-line UV detector PN3211 (PostNova Analytics, Denver, CO). Figure S5 shows substantial resolution of the 30S and 50S ribosomal units and the 70S ribosome complex. The hydrodynamic diameters calculated for these three observed populations (16.9, 20 and 22.7 nm for the 30S, 50S and 70S units respectively) agree with literature values.

**Figure S5.** Field flow fraction separation analysis of a 1:2 dilution of the cell-free extract showing tRNA and ribosomal subunits measured at 260 nm. Ribosome and subunit concentrations were determined using the extinction coefficients: 30S - 1.3×10^7^, 50S - 2.7×10^7^, 70S - 4.0×10^7^.

**References**

Ahn JH, Kang TJ, Kim DM. 2008. Tuning the expression level of recombinant proteins by modulating mRNA stability in a cell-free protein synthesis system. Biotechnol Bioeng 101(2):422-7.

He B, Rong M, Lyakhov D, Gartenstein H, Diaz G, Castagna R, McAllister WT, Durbin RK. 1997. Rapid Mutagenesis and Purification of Phage RNA Polymerases. Protein Expression and Purification 9(1):142-151.

Kim DM, Swartz JR. 2004. Efficient production of a bioactive, multiple disulfide-bonded protein using modified extracts of Escherichia coli. Biotechnol Bioeng 85(2):122-9.

Olins PO, Devine CS, Rangwala SH, Kavka KS. 1988. The T7 phage gene 10 leader RNA, a ribosome-binding site that dramatically enhances the expression of foreign genes in Escherichia coli. Gene 73(1):227-35.

Takeshita S, Sato M, Toba M, Masahashi W, Hashimoto-Gotoh T. 1987. High-copy-number and low-copy-number plasmid vectors for lacZ[alpha]-complementation and chloramphenicol- or kanamycin-resistance selection. Gene 61(1):63-74.

Zhang S, Krivosheyeva A, Nochumson S. 2003. Large-scale capture and partial purification of plasmid DNA using anion-exchange membrane capsules. Biotechnol Appl Biochem 37(Pt 3):245-9.
